# Supplementary material for: Patterns of Intron Gain and Loss in Fungi
Source: PLoS Biol. 2004 Nov 30;2(12):e422. doi: 10.1371/journal.pbio.0020422 (PMC532390; doi:10.1371/journal.pbio.0020422)
Supplement: Table S1 — Also available at http://genes.mit.edu/NielsenEtAl/. (4.3 MB ZIP). [file pbio.0020422.st001.zip › NielsenEtAl/html/341.html]

AN6506.1.NCU06207.1.MG08832.1.FG02502.1


```
 CLUSTAL W (1.82) Multiple Sequence Alignments - Introns Inserted


Sequence 1: AN6506.1	352 aa
Sequence 2: MG08832.1	344 aa
Sequence 3: NCU06207.1	344 aa
Sequence 4: FG02502.1	349 aa
Alignment Length: 353 aa
Number Identitical Residues: 153 aa
Alignment Score (without introns) 8889


MG08832.1 	MDIVLEITDTFLWDYMYAFVAPA----TAPPYDYPNLSNGTAVAHNG---WEYKPTTALF
NCU06207.1	MDVVLEVTDQFMFDYMYAWLLPA----RPALYDFPDKTNGTAQAFSS---WVYEPATKFF
FG02502.1 	MDVVLEVVDTFIADYAYAYFYPK----RLAPYDFPSPSNTTDTSAKAFSTWIYKPATQFI
AN6506.1  	MDIALELFDTFVGDRLYSALLPASLSSTVSVPGFPTAANSTLSLFGASKPFVYEPATKLL
          	**:.**: * *: *  *: . * : ::  .  .:*  :* *     . ..: *:*:* ::

MG08832.1 	SLEPSQAAYQSALPRDNIYRQGISLFLIYW2IFGVLVYYIFATASYLFIFDKKQMEHPKF
NCU06207.1	SLEPSQAAYQSIWTRDNIYRQALSLFLILW2LFGLVTYYVFASLSYIFVFDKKTMEHPKF
FG02502.1 	TLEPPEQAYMSSWDRDNPLRQALTLYLITW2IFGLLVYFIVATLSYIFIFDKRTFEHPRF
AN6506.1  	YLEPSKYAYLSAWPRNNIYRQFLSFFLIVW2IFGFIVYFISATLSYMFIWDKSTVKHPKF
          	 ***.: ** *   *:*  ** ::::** * :**.:.*:: *: **:*::**  .:**:*

MG08832.1 	LKNQIKLEMIQANKAFPIMALYTVPMILLEIRGYSKLYDSADEAPAWWYNYIQFPLFLVF
NCU06207.1	LKNQVWLEIKQTNAALPVMAFFTFPFLVAEVRGYSLLYDTTAEGPGRWYDFFQFPLFIMF
FG02502.1 	IKNQVRMEIIAANKAMPVMAIITAPFFLLEVQGYGKLYDTTEDGPGLWYDFFQFPLFLLF
AN6506.1  	LKNQIPLEIAQTMRSMPIMSLLTAPFLVAEVRGYAKLYDTFDEEPFPYYSILQFPLFIAF
          	:***: :*:  :  ::*:*:: * *::: *::**. ***:  : *  :*. :*****: *

MG08832.1 	TDFCIYWIHRWLHAPIFYKRLHKPHHKWIMPTPFASHAFHPLDGYAQSFPYHLFPFIFPL
NCU06207.1	TDFGIYWIHRGLHHPLVYKHLHKPHHKWIMPTPYASHAFHPIDGFAQSIPYHIFPFIFPL
FG02502.1 	TDFCIYWAHRWLHHRLVYKYLHKLHHKWIMPTPFASHAFHPLDGFTQSLPYHIFPFIFPL
AN6506.1  	TDFFIYWIHRGLHHPLVYKTLHKPHHKWIMPSPFASHAFHPVDGWSQSVPYHVFPFIFPL
          	*** *** ** **  :.** *** *******:*:*******:**::**.***:*******

MG08832.1 	QKVAYIGLFVFINIWTILIHDGEYVANNPIINGAACHAVHHFAFNYNYGQYTTLWDRLGG
NCU06207.1	QKMAYVGLFVFINFWTIMIHDGEYYANNPVINGAACHSVHHFAFNYNYGQFTTLWDRLGG
FG02502.1 	QKMAYVALFVFVNLWSVMIHDGEYLTNNPVVNGAACHSLHHSRFEVNYGQFFTAFDRMGG
AN6506.1  	QKAAYVLLFGFINIWTVMIHDGEYVANSPVINGAACHTMHHLYFNYNYGQFTTLWDRMGG
          	** **: ** *:*:*:::****** :*.*::******::**  *: ****: * :**:**

MG08832.1 	SYREPDADLFDLKAKMSKEQWNKQAKEMENLVQQIEGEDDRVYGEISDKKTR--
NCU06207.1	SYREPDGDMFAKEKKMSTTTWKKQVNEMEKIVKEVEGEDDRLYEPTETKKSK--
FG02502.1 	TYRMPEQWMFERDMKMSEGRWKKEIEKVDELIEEIEGSDNRTYTDSAPIMKKTQ
AN6506.1  	SYRKPNEELFRRETKMGEEEWKRQTKEMETILKDVEGDDDRKYLAEEDSKKNL-
          	:** *:  :*  . **.   *::: :::: :::::**.*:* *       ..
```
